# Modeling omics dose-response at the pathway level with DoseRider

**DOI:** 10.1016/j.csbj.2025.04.004

**Published:** 2025-04-03

**Authors:** Pablo Monfort-Lanzas, Johanna M. Gostner, Hubert Hackl

**Affiliations:** a Institute of Medical Biochemistry, Biocenter, Medical University Innsbruck, 6020 Innsbruck, Austria; b Institute of Bioinformatics, Biocenter, Medical University Innsbruck, 6020 Innsbruck, Austria

**Keywords:** Benchmark dose, Dose-response modeling, Mixed models, Multi-omics, System biology, Trend change dose, Toxicology

## Abstract

The generation of omics data sets has become an important approach in modern pharmacological and toxicological research as it can provide mechanistic and quantitative information on a large scale. Analyses of these data frequently revealed a non-linear dose-response relationship underscoring the importance of the modeling process to infer biological exposure limits. A number of tools have been developed for dose-response modeling and various thresholds have been defined as a quantitative representation of the effect of a substance, such as effective concentrations or benchmark doses (BMD). Here we present DoseRider an easy-to-use web application and a companion R package for linear and non-linear dose-response modeling and assessment of BMD at the level of biological pathways or signatures using generalized mixed effect models. This approach allows to analyze custom or provided multi-omics data such as RNA sequencing or metabolomics data and its annotation of a collection of pathways and gene sets from various species. Moreover, we introduce the concept of the trend change doses (TCDs) as a numerical descriptor of effects derived from complex dose-response curves. The usability of DoseRider was demonstrated by analyses of RNA sequencing data of bisphenol AF (BPAF) treatment of a human breast cancer cell line (MCF-7) at 8 different concentrations using gene sets for chemical and genetic perturbations (MSigDB). The BMD for BPAF and a set of genes upregulated by estrogen in breast cancer was 0.2 µM (95 %-CI 0.1–0.5 µM) and the lowest TCD (TCD1) was 0.003 µM (95 %-CI 0.0006–0.01 µM). The comprehensive presentation of the results underlines the suitability of the system for pharmacogenomics, toxicogenomics, and applications beyond.

## 1 Introduction

In many different settings, the effects of compounds or drugs are studied in different healthy or disease models at the cell or organism level. Thereby the dose at which a specific effect, change in phenotype, or apical outcome such as mortality can be observed is of particular interest. The consideration of dose–response plays an important role in human risk assessment. It is meanwhile recognized that certain chemicals such as endocrine disruptors can have particularly harmful effects at low doses and that non-monotonic dose responses are common [1]. In addition, new concepts such as hormesis, toxicological thresholds of concern (TTCs), and dose-dependent transitions in toxicity mechanisms were introduced, highlighting the complexity associated with the characterization of dose-response relationships [2], [3]. These are in contrast to the assumption that high dose hazards can be used to predict low dose safety, and this insight has far-reaching consequences on default procedures for dose extrapolation in risk assessment [4].

The generation of omics data sets has become an important approach in modern pharmacological and toxicological research as it can provide mechanistic and quantitative information on a large scale and allow to study the dose response at the molecular level. Broader recognition of the utility of toxicogenomics (and especially transcriptomics) data for human health protection is clearly warranted [5], also in light of the emerging importance of the application of adverse outcome pathways (AOP) and new approach methods (NAMs) for risk assessment [6]. Regulatory agencies such as the National Institute of Environmental Health Sciences, in cooperation with the National Toxicology Program (NTP) and US Environmental Protection Agency (EPA) has initiated the development of approaches and tools using these data to define potential mode of actions and to gain mechanistic understanding of adverse events. Specifically, BMDExpress [7] and successors BMDExpress 2 and 3 [8] allow to analyze toxicogenomics data and assess the risk or define regulatory thresholds. The online tool FastBMD is another example which allows a rapid determination of the benchmark dose (BMD) based on transcriptomics dose response data [9]. The EPA's Transcriptomic Assessment Product (ETAP) for pathway-level assessment of gene sets or points of departure (PoD) suggests using the mean BMD or BMDL value as the relevant threshold [10]. Analyses of molecular responses or concentration-response curves often show a sigmoidal trend and are considered monotonic, but this is not necessarily the vast majority as some studies indicate [11], [12]. Corresponding analyses include the fitting of sigmoid-like parametric functions (for example log-logistic, Weibull, Hill, or Gamma function) but can also be extended to biphasic function categories (U-shaped, bell-shaped) and calculated using commercial software packages such as GraphPad (http://www.graphpad.com). Once a function representing the dose-response relationship is fitted, different parameters like the sensitivity threshold (benchmark dose), points of departure, or the median effective concentration (EC50) can be robustly and precisely estimated [13]. More recently, R based packages and workflows for dose-response analysis are available, for example *ToxicR* [14], *drc* [15], the Toxcast pipeline *tcpl* [16]*, tcplfit2* [17]*,* and *PROAST* [18]*.* Developments for multi-omics data analyses such as the R based tool *DRomics* [19] as used for ecological risk assessment has exemplified that the dose-response framework may be applied to other types of omics-data e.g. proteomics and metabolomics data. Interestingly, this approach allows to assess the cumulative distribution of sensitives of features (BMD) across GO (Gene Ontology) terms or KEGG (Kyoto Encyclopedia of Genes and Genomes) biological pathways. Although not designed for dose-response analysis, Multi-Gene Set Enrichment Analysis (*MultiGSEA*) [20] allows to detect the activation of pathways across different omics-datasets at specific compound concentrations. Building on GSEA approaches, some methods use leading-edge genes from GSEA analysis to focus on the most biologically meaningful genes when computing pathway-level BMDs [21]. Other approaches provide a broader pathway-level perspective. For example, some methods apply dimensionality reduction techniques, such as PCA or latent variable models, to derive dose-response relationships [22], [23]. More recently, Bayesian gene set benchmark dose (BS-BMD) has been proposed as a novel approach for pathway-level point-of-departure estimation [24].

However, there is still a need to determine dose-responses at pathway level and to extract quantitative information of complex dose-response curves beyond the BMD concept. Therefore, we have developed and present here DoseRider, which is an easy-to-use web application and a companion R package for dose-response modeling. This approach includes several features (1) using mixed effect models allow to fit a non-linear function (not limited to predefined functional classes) for each feature, (2) to fit a dose-response model for a pathway (gene signature, set of features) as a whole, (3) to assess sensitivity threshold at the pathway level, (4) application on different omics-data and for a number of species. We detail all dose response modeling steps and the web server interface and will demonstrate its utility in a toxicogenomics case study based on RNA sequencing data.

## 2 DoseRider features and methods

### 2.1 Implementation

DoseRider is a web application (https://doserider.i-med.ac.at) and a companion R package designed for dose-response modeling at the pathway level. The web application is built using the Django framework (version 4.2) and utilizes Django_rq (version 2.7.0) for queuing tasks due to the computationally intensive nature of the analyses. PostgreSQL (version 12.0) serves as the database management system, handling the information, results, dataset, queues, and gene sets. Nginx functions as a reverse proxy, directing client requests to Gunicorn, which serves the Django application while also managing static files. The graphical user interface is implemented using Bootstrap 4, with all plots generated via the *ggplot2* R package. The entire DoseRider environment is encapsulated within a Docker image (version 5.0.0), with Docker Compose (version 1.29.2) facilitating the integration of all components (https://hub.docker.com/r/icbi/doserider). DoseRider is readily accessible online without requiring any login, allowing users to view all public analyses. Anonymized user data is automatically removed from the server after seven days, ensuring privacy. The platform offers a manual that includes an example dataset for users to explore. The backend analysis is performed using the developed DoseRider package implemented in R (https://www.R-project.org, R Foundation for Statistical Computing, Vienna, Austria). The DoseRider R library code is open-source, licensed under the MIT License, and can be accessed via the GitHub repository https://github.com/icbi-lab/doserider. The generalized mixed effect models were fitted using the *lmer* function from the *lme4* R package. For RNA sequencing data, dispersion from a negative binomial distribution was calculated using the *edgeR* package [25]. Since the fitting process is computational expensive, an option for parallelized processing on a computer cluster was implemented in the DoseRider R package.

### 2.2 Data sets

The primary goal of DoseRider is to model pathway expression or intensity data from dose-response experiments, particularly focusing on nonlinear relationships using mixed models with cubic splines. This analysis requires comprehensive pathway information, including gene sets, annotations, and features such as genes or metabolites. To achieve this, DoseRider utilizes datasets sourced from various well-established repositories. Key pathway, hallmarks, and gene set information was sourced from the Molecular Signatures Database (MSigDB) [26], which supports multiple species such as *Homo sapiens*, *Mus musculus*, *Rattus norvegicus*, *Drosophila melanogaster*, *Danio rerio*, and *Caenorhabditis elegans*. These gene sets were downloaded using the *msigdbr* library in R. Additionally, other relevant pathway data were integrated, including immune related gene signatures [27] and data from the ConsensusPathDB [28]. In addition, we identified a collection of significantly affected pathways (high-response toxicogenomics gene sets) across treatments present in the Toxicogenomics Project-Genomics Assisted Toxicity Evaluation System (TG-GATES) database [29], containing data from 146 compounds belonging to different chemical classes. We performed Gene Set Enrichment Analysis (GSEA) on differentially expressed genes for each compound and dose level compared to the respective control. A score for each pathway was calculated by averaging the individual scores (log10 (p-value)*normalized enrichment score (NES)) across all compounds and dose levels. In addition, a list of enriched gene sets for each of the 146 compounds was provided. These curated gene sets emphasize the most responsive genes involved in toxicological responses, facilitating a targeted approach in dose-response analysis. However, users are not restricted to use these gene sets. DoseRider allows full flexibility in selecting gene sets from the pre-integrated databases or uploading custom pathway files in GMT format as commonly used for GSEA.

DoseRider expects two primary input files (1) an expression data file and (2) a metadata file. The expression data file should contain raw counts from RNA sequencing experiments or log2-intensity values from microarray, proteomics or metabolomics experiments. The metadata file should provide information about the doses administered to each sample, with matching sample identifiers between the two files. In *in vitro* experiments, the nominal concentration in the test media are commonly used as surrogate dose to describe the effect (or no-effect) doses [30]. In order to estimate such dose/concentrations for a cellular response, for example half maximal effective concentrations, EC50 values, data from relevant biological responses such as viability, cytotoxicity, proliferation, or mitochondrial activity can be included.

To demonstrate the capabilities of DoseRider several public datasets have been downloaded, preprocessed, and are available at the DoseRider web application. These include RNA sequencing data from experiments involving 16 bisphenol and bisphenol alternative chemicals at various concentrations [31], microarray data detailing transcriptional responses to four kinase inhibitors across a five-logarithm dose range [12], and metabolomics data from BPA dose-response experiments [32]. Each dataset was preprocessed in accordance with the methodologies described in their respective publications.

### 2.3 Workflow

**Fig. 1:**
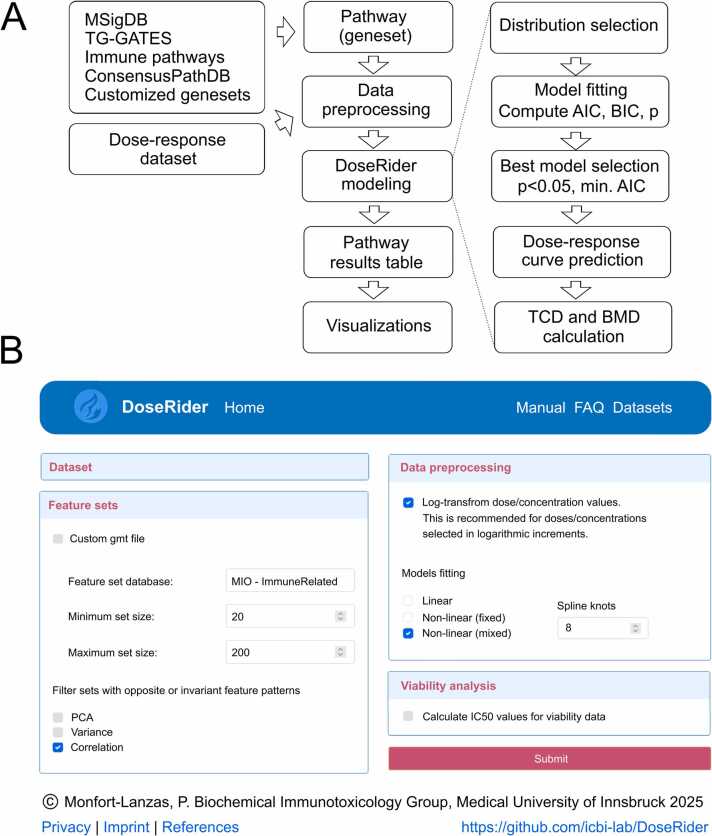
Information about the DoseRider web application for dose-response modeling. A) Workflow starting with a dose-response dataset and pathway and various steps during the modeling process. B) Details of the web interface for selecting the feature (gene) sets (which could be pathways, gene ontology terms, or any set of features). Note that there is the option to filter sets showing divergent response curves. MSigDB, Molecular signature database; TG-GATES, Toxicogenomics Project-Genomics Assisted Toxicity Evaluation System; AIC, Akaike information criterion; BIC; Bayesian information criterion; IC50, half-maximal inhibitory concentration; TCD, trend change dose; BMD, bench mark dose; PCA, principal component analysis.

The DoseRider analysis workflow is designed to assess dose-response relationships in pathway expression data using generalized linear mixed model (GLMM) and extension for non-linear relationship with cubic splines [33]. This approach is particularly suited for omics data, such as RNA sequencing or microarrays, where the objective is to model gene expression across varying dose levels of an exposure compound. DoseRider workflow includes steps for data pre-processing, model fitting, significance testing, smoothing of response curves, and calculation of Benchmark Doses (BMDs) (Fig. 1A). DoseRider offers the access to several pre-processed datasets or expression data file together with a meta datafile, the omics type, species, and feature identifier (e.g gene symbol) can be uploaded, pre-processed and link to pathway or gene set information, which can be also upload as custom file in GMT format. The user can customize the analyses by modifying the (1) number of features in the set, (2) apply optional filtering steps to exclude pathways including non-informative or highly antagonistic genes, (3) build clusters for divergent patterns within the pathway (this is automatically calculated in the background), or (4) log10- transformation doses when the range of the doses are widely distributed (Fig. 1B).

Pathways could be filtered for invariant genes when more than 50 % of genes in the pathway had a variance within the 10 % quantile of lowest variances among all genes, or opposite gene profiles using the percentage of pairwise negative correlation of all genes in the pathway is higher than 50 %, or if the first principal component explains more than 70 % of the variance. DoseRider allows to change these default cutoff values.

After model fitting the output of the DoseRider analysis includes a comprehensive summary for each pathway, highlighting the best-fitted model along with relevant information such as AIC values, p-values, BMD estimates, and estimated dose-response curves. These results are compiled into a table that can be downloaded in various formats. Additionally, the DoseRider results can be saved for further processing directly in R. The web server also allows for the generation of various plots, enabling in-depth characterization of the dose-response relationships.

### 2.4 Model fitting

For each gene set, DoseRider fits a series of generalized linear mixed models (GLMM) to evaluate the dose-response relationship, enabling the modeling of pathway-level dose-response dynamics (Eq. 1). The models considered include (1) a null model, which represents no dose-response relationship and serving as a baseline for comparison, (2) a linear mixed model assumes a linear relationship between dose and gene expression, with dose as the fixed effect and gene-specific random intercepts and slopes (Eq. 2), (3) a non-linear fixed model incorporates non-linear dose-response relationships using cubic splines for the fixed effects (dose), and (4) non-linear mixed model extends the non-linear fixed model by adding gene-specific cubic splines (Eq. 3) allowing for the accurate representation of gene-specific variations and providing a comprehensive understanding of how entire pathways respond to different doses. The general model formulation is:

(1) $$Y_{\mathrm{ij}}=\beta_{0}+b_{0j}+f_{j}\left(D\right)+\epsilon_{\mathrm{ij}},$$

where $Y_{\mathrm{ij}}$ is the expression of gene j in sample i. $\beta_{0}$ is the global intercept (average expression of the pathway), $b_{0j}$ is the gene-specific random intercept, capturing variation in baseline expression between genes. $f_{j}\left(D\right)$ is the dose-response function, that can be linear or non-linear approximated by cubic splines. For the linear function applies

(2) $$f_{j}\left(D\right)=\left(\beta_{1}+b_{1j}\right)D$$

where $\beta_{1}$ is the fixed dose effect (shared across genes), $b_{1j}$ is the gene-specific random slope, allowing each gene to deviate from the global dose-response. The non-linear function is approximated by cubic splines:

(3) $$f_{j}\left(D\right)=\sum_{k=1}^{K+d+1}\left(\beta_{k}+b_{\mathrm{kj}}\right)N_{k}\left(D\right)$$

where $N_{k}\left(D\right)$ are the basis functions of the cubic spline, $\beta_{k}$ represents the fixed effect of the spline basis functions, $b_{\mathrm{kj}}$ represents gene-specific random deviations in the spline coefficients, K is the number of knots, and d is the degree (d = 3 for cubic splines). For spline based models, the number and placement of knots are corresponding to the different concentrations but can be selected manually for each of the concentrations.

In order to determine linear or non-linear trends this modeling approach allows to select the best model for each gene set with lowest Akaike information criteria (AIC) and a significant p-value (p < 0.05 or if selected otherwise) compared to the null model using likelihood ratio test. To better understand the trends in gene expression across doses, the selected best-fit model is used to generate the predicted dose-response curve. To elucidate genes with similar dose-response patterns *k*-means clustering is performed. This step is optional and is particularly useful when gene sets exhibit divergent responses to the exposure. The optimal number of clusters (*k*) is determined using the silhouette method [34], and each cluster is analyzed separately to identify distinct response patterns. This clustering provides additional insights into the biological processes affected by the exposure and helps to identify subsets of genes with similar dose-response patterns.

### 2.5 Calculation of benchmark dose (BMD) and trend change dose (TCD)

The BMD is an important indicator in the assessment of chemical risks, as it can be used as a point of departure (POD) to derive human health guidance values. A BMD is a dose or concentration that produces a predetermined change in the response rate of an adverse effect, the benchmark response (BMR) with default values of 5 % or 10 % change in the response rate relative to control (https://www.chemsafetypro.com). In toxicogenomics, the BMD represents the dose at which a specific level of gene expression change is observed [7]. DoseRider computes BMD values for each gene set based on the smoothed dose-response curves. The BMD is calculated using y0 + /- z * SD, with y0 is the level at the control given by the dose-response model, SD is the residual standard deviation of the dose response model fit and z is derived from the chosen benchmark response (BMR), with a default of 1 (corresponding to a one-sided BMR of 10 %) but adjustable by the user [35]. For biphasic dose-response curves, where gene expression increases at lower doses and decreases at higher doses (or vice versa), the BMD is defined as the lowest dose at which the BMR is observed. The 95 % BMD confidence interval were analysed using 1000 times bootstrap with 60 % of data. In addition, we defined the trend change doses (TCD) as doses with a maximum, minimum or inflection point of the dose-response curves and include a 95 % confidence interval derived from 1000 times bootstrap using 60 % of data. TCD1 indicate the TCD at the lowest dose/concentration.

## 3 Results and discussion

Omics data have contributed to gaining insights into the complexity of dose-response relationships, which are of utmost importance for understanding the mode of action of chemicals or drugs and thus ultimately on the protection of human health. The resulting ability to model nonlinear responses in an unbiased way, so that they are not neglected in the analysis of relationships associated with an adverse outcome, has important implications for the development and discussion of concepts for health-based guidance values. Taking this into consideration, we developed DoseRider and illustrated its workflow and analytic effectiveness using data from a dose-response analysis of bisphenol AF (BPAF) on human breast cancer cell line (MCF-7), treated with a range of doses (0.0005–10 µM). To accommodate the wide range of doses, the dose values were log10-transformed for the analysis. The analyses were performed using a collection of chemical and genetic perturbation gene sets from MSigDB. For each pathway, the best-fitting model was selected based on a significantly different response compared to the null model (p < 0.05; likelihood ratio test) and the minimum Akaike information criterion (AIC). All significant pathways, along with their respective parameters, are summarized in Table 1.

**Table 1:** Significant genesets from DoseRider analysis of BPAF treatment of human breast cancer cell line (MCF-7) with parameters of the best fitted models and information on benchmark dose.

Geneset	ID	Best model fit				BMD	
		Best Model	*P*	Adj. *P*	AIC	Median	95 % CI*
IGF2BP1 targets	M42507	NLMM	<0.001	<0.001	11,378	0.061	0.030–0.127
Breast cancer ESR1 up	M393	NLMM	<0.001	<0.001	16,921	0.190	0.089–0.492
Dasatinib resistance dn	M16369	NLMM	<0.001	<0.001	7374	0.186	0.003–0.600
PARVB targets up	M2238	NLMM	<0.001	<0.001	9680	0.639	0.012–6.468
BRCA metaplastic vs ductal dn	M737	NLMM	<0.001	<0.001	8716	0.084	0.002–1.676
Glioblastoma proneural	M2115	NLMM	<0.001	<0.001	7106	0.164	0.050–0.603
Tumor suppression by SMAD1 dn	M2186	NLMM	<0.001	<0.001	12,352	0.147	0.044–0.672
Hypoxia up	M7363	NLMM	<0.001	<0.001	13,376	0.191	0.050–0.730
SDHB targets up	M15549	NLMM	<0.001	<0.001	7281	0.183	0.033–0.818
Wilms tumor up	M12621	NLMM	<0.001	<0.001	16,580	0.295	0.033–3.536

* 95 %-CI, BMD 95 % confidence interval from 1000 times bootstrap analysis; AIC, Akaike information criteria; BMD, benchmark dose; ID, database identifier in MSigDB; *P*, p-value from likelihood ratio test against the null model; Adj. *P*, adjusted p-value based on FDR according to the Benjamini-Hochberg method; NLMM, non-linear mixed model.

**Fig. 2:**
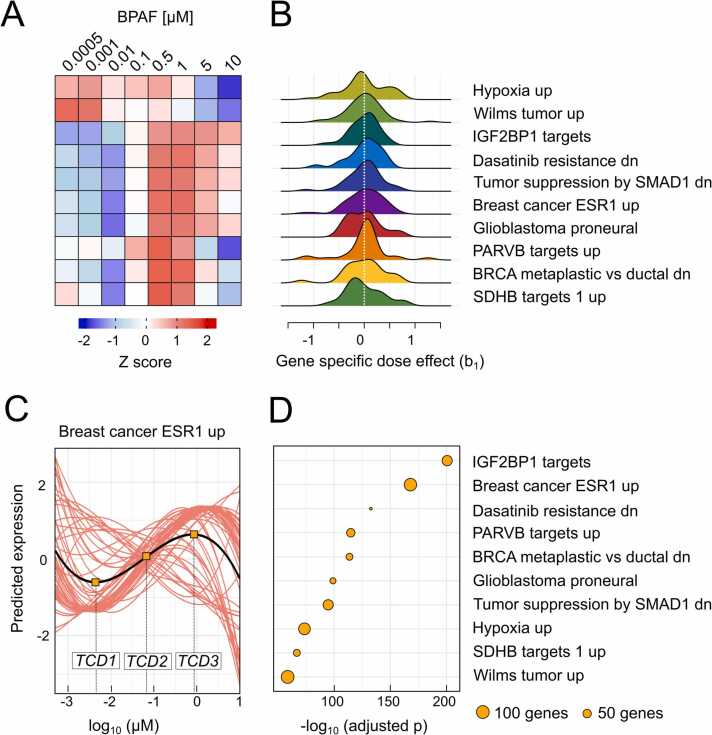
Visualization of results from dose-response analyses by DoseRider of bisphenol AF at various concentrations in the human breast cancer cell line (MCF-7) for gene sets from the MSigDB collection (chemical and genetic perturbations). A) Heatmap of z-score of the pathways with the best fit (minimum AIC) indicating activation at 0.5 µM. B) Distribution of gene specific effects indicate similar patterns of genes. C) Dose response of genes upregulated in breast cancer by estrogen, computed trend change doses (TCD) are indicated by orange boxes (TCD1 = 0.003 µM, TCD2 = 0.06 µM, TCD3 = 0.8 µM). The black line indicate the fitted pathway-level trend, derived from the mixed model, the red lines indicate fitted gene expression level trends. D) Significant non-linear dose dependence of selected pathways (size indicate number of genes, position to the right indicate higher significance (-log10 (adjusted p-value)).

Using DoseRider, we were able to identify several pathways significantly affected by BPAF exposure, underscoring its potent endocrine-disrupting effects [36]. The heatmap from the top significant pathways (Fig. 2A) illustrates dose-dependent gene expression changes across a range of BPAF concentrations, with early transcriptional shifts evident even at low doses. Fig. 2B shows the distribution of the gene specific dose effects for each pathway, allowing the assessment of variability of dose-response across genes. Wider or multimodal distributions indicate more diverse gene responses, while narrower distribution suggest a more consistent response. As one of the most central finding we detected a non-linear dose-response of BPAF for the *Breast cancer ESR1 up* gene set displaying non-monotonic activation across concentrations (Fig. 2C). This behavior is typical of endocrine disruptors, where gene expression does not increase linearly with dose, a well-documented phenomenon in estrogenic compounds like BPAF [37]. BPAF promotes the proliferation of ER-positive breast cancer cells by activating both the ERα and ERβ, which are nuclear receptors and regulate the transcriptional activity of several targets, which are included in this gene signatures [36]. Further highly significant key pathways comprise *IGF2BP1 targets* and *PARVB targets* (Fig. 2D). There is a known crosstalk between estrogen receptors and growth factor signaling, specifically IGF, enhancing tumorigenic potential and could explain the pronounced effects [38], [39]. PARVB is implicated in cell adhesion, migration, and cytoskeletal organization, suggesting that BPAF may also influence metastatic potential by altering this pathway [40].

**Fig. 3:**
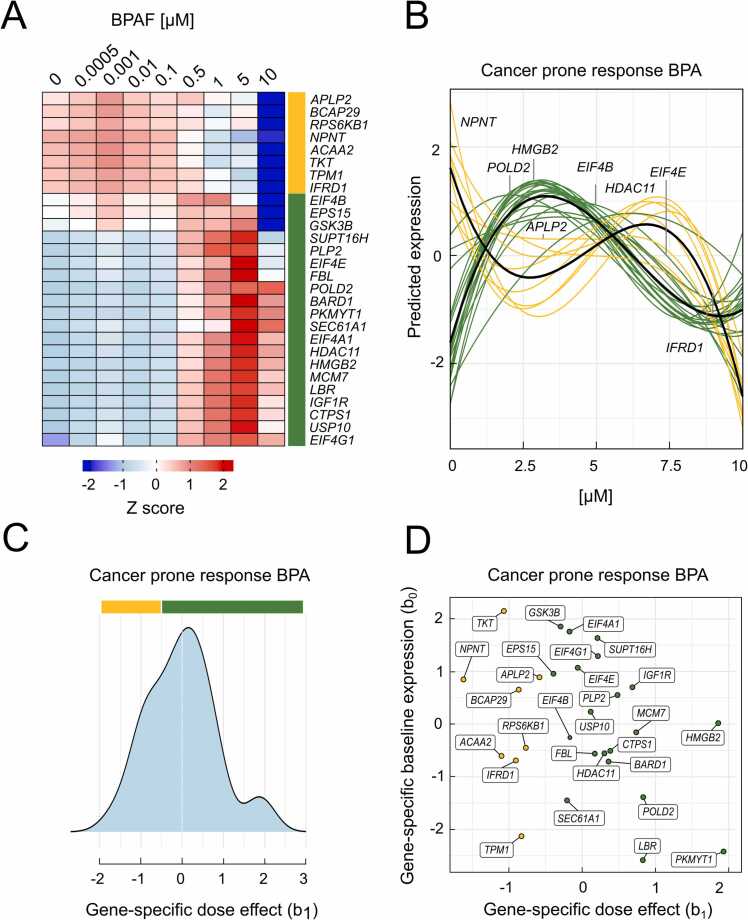
Dose response profiles for individual genes in the *Cancer prone response BPA* pathway. A) Heatmap of z-score from gene expression at different bisphenol AF concentrations. Genes have been optimally clustered into two groups with different expression profiles. B) Dose response curves (predicted expression from the fitted non-linear models) of genes in the two clusters (green and yellow). Trend of expression is indicated separately for each cluster (black). C) Distribution of gene-specific dose effects. D) Clustering of genes based on gene-specific dose effect (b_1_) and gene-specific baseline expression (b_0_).

Dose-response modeling with DoseRider not only determines a representative dose-response curve for each pathway, but also provides a dose-response pattern for each individual gene. However, the analysis of the *Cancer Prone Response BPA* set showed that the genes exhibited divergent dose-response behavior upon BPAF exposure (Fig. 3). DoseRider can perform clustering analyses and group features with similar dose-response pattern into a cluster. The most dominant cluster including the most features could be potentially best represent the overall changes of a pathway. In the *Cancer Prone Response BPA* pathway genes were clustered into two groups highlighted in Fig. 3A, B. The distribution of the gene specific effects from the mixed effects model are summarized in Fig. 3C, D. Many genes from the dominant cluster (Fig. 3, indicated in green) are involved in cell cycle and processes that, when dysregulated, can promote cancer development, cell proliferation, and survival. Key components of the eIF4F complex—EIF4E, EIF4G1, and EIF4A1—are crucial for cap-dependent translation initiation. Overexpression of these factors, frequently observed in cancer, is linked to increased protein synthesis and uncontrolled cell proliferation [36]. The strong activation of genes from the dominant cluster such as *EIF4E*, *MCM7*, and *IGF1R* at low BPAF concentrations (0–2.5 μM) suggests that even at relatively low doses BPAF can potentially enhance cancer cell growth (Fig. 3B).

**Fig. 4:**
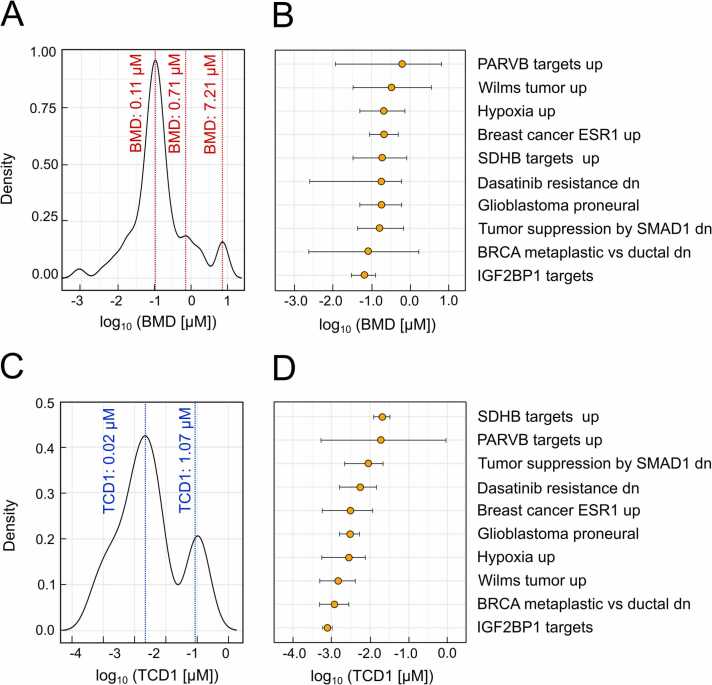
Summary of benchmark doses for bisphenol AF in human breast cancer cell line (MCF7). A) Cumulative benchmark doses over all significantly affected pathways. B) Median benchmark doses for significantly affected pathways and 95 % confidence interval from 1000 bootstrap analyses using 60 % of data. C) Density of cumulative first (lowest) trend change doses (TCD1) over all significantly affected pathways. D) Median TCD1 for significantly affected pathways and 95 % confidence interval from 1000 bootstrap analyses using 60 % of data. All concentrations were log_10_ transformed.

Omics datasets can also be used to compare the strength of the effect of different substances on specific responses and cellular processes with other parameters such as cytotoxicity data [12]. A major improvement of DoseRider is to assess the BMD at the pathway level, that is not only summarize results from individual genes but to model the pathway/gene set as a whole and derive effective concentrations. Using a bootstrap method in DoseRider helps to estimate the 95 %-confidence interval of the pathway BMD. BPAF exhibited significant biological effects at low concentrations (0.11 µM) across various pathways (Fig. 4A). This is in line with previous studies identifying BPAF as one of the most potent bisphenol analogs [17], [21]. Pathway-specific BMD estimates were highlighted in Fig. 4B and Table 1. While the BMD for the most significant pathways was 0.1 µM, the lower BMD limits (BMDL) reached only 0.002 µM, underlining the potency of BPAF. This is consistent with previous research, showing that BPAF at concentrations as low as 0.001 µM to 1 μM significantly increased cell viability, further underscoring the relevance of these low-dose effects [41]. The concept of the BMD actually comes from toxicological risk assessment where the BMD describes the dose or concentration associated with predetermined change in the response rate of an adverse effect and is used as a point of departure (POD) to derive human health guidance values. However, this concept does not take into account more complex dose-response relationships. Therefore, we have introduced the concept of the trend change doses (TCDs) as a numerical descriptor of especially non-linear dose-response relationships indicating points of deviation from the trend. These include maximum, minimum, or inflection points in the estimated dose-response curves and could represent first indicators for changes of cellular processes. For example in the *Breast cancer ESR1 up* the three TCDs upon BPAF exposure, TCD1 = 0.003 µM, TCD2 = 0.06 µM, and TCD3 = 0.8 µM can be clearly derived (Fig. 2C). Importantly, when we compare overall BMD levels with TCD1 levels (Fig. 4) it turned out that TCD1 distribution is almost an order of magnitude lower than the BMD distribution indicating that effects can be observed already at lower concentrations. The TCD concept provides a powerful mean to automatically generate descriptors of the effects on transcript/pathway responses, thus enabling a comparative analysis with ECs obtained by standard cellular assays.

The comparison of the estimated dose-response curves with DoseRider with the actual readout of the respective omic layer proved to be a reasonable modeling as outlined in Figure S1 for selected genes of pathways significantly affected by BPAF or BPA. As goodness-of-fit measure the root mean square error (RMSE) is provided and ranged from 0.53 to 1.39 for a number of *IGF2BP1 target* genes from RNA sequencing data (Figure S1A) and for selected metabolites of the *Tryptophan metabolism* pathway for BPA from 0.14 to 0.17 (Figure S1B).

As the analyses of the BPAF dose response demonstrate DoseRider has not only many option for fitting non-linear dose-response models and provide comprehensive visualization but it is also able to estimate reasonable BMDs for various processes. However, DoseRider has several limitations. For instance the fitting models can be computationally intensive and time-consuming. For this reason DoseRider allows parallel distribution of computations on a computer cluster. Currently our application lacks the capability to perform multi-omics analyses in one step, meaning it cannot integrate and model different omics layers (such as transcriptomics, proteomics, and metabolomics) simultaneously but ongoing efforts will support the integration of pathway information across omics entities. Another important factor is the minimum number and distribution of doses used in the analysis. Previous studies have shown that more points improving parameter estimation and reliability and at least four doses are recommended for fitting cubic splines, while three are sufficient for linear trends [42], [43].

A key challenge in pathway-level modeling is the presence of antagonistic gene expression patterns within a gene set. DoseRider addresses this through optional filtering steps removing pathways with this type of opposite patterns. In addition, DoseRider provides the possibility to detect gene subgroups with distinct patterns by clustering and resulting gene list from each of the clusters can be saved as new gene sets. However, in some cases elements have different impact on the pathway response, but weights of individual elements are not considered in the modeling.

We introduce the concept of trend change doses (TCDs), which identify specific doses at which the direction of the nonlinear dose-response curve changes. TCDs are a complementary concept to BMD, which quantify risk based on predefined adverse effect levels (e.g., 5–10 % response change) [35]. The advantage of TCDs is that they react more sensitively to signal changes and reveal earlier biological transitions. Not all of these transitions reflect adverse effects, but may indicate critical events in a cell that can, for example, trigger compensatory mechanisms to return to homeostasis. In addition, knowledge on such early events can reveal various processes and targets, which offer opportunities for preventive treatment strategies [44], [45], [46], [47]. The TCDs therefore contribute to the comprehensive description of the MoA of a compound.

DoseRider provides a key advantage over existing methods by using predefined gene sets and modeling all genes within a pathway simultaneously with mixed models and cubic splines. In contrast, tools such as FastBMD [9] and DRomics [19] fit a limited number of functions (e.g., linear, polynomial, Hill) to each gene individually. Then they compute the BMD for each gene, and derive the pathway-level BMD by aggregating these values using confidence intervals or percentile-based estimates. Other approaches that work at the pathway level, such as those applying PCA or latent variables to reduce dimensionality before modeling [22], [23], still rely on transforming the data rather than modeling the entire pathway directly. An interesting approach is the Bayesian gene set benchmark dose estimation [24], which computes a pathway-level BMD by incorporating the prior distribution of gene responses and their correlations within a gene set but does not focus on modeling the pathway dose-response pattern. Recently also deep machine learning algorithms become available but were mostly used to analyze dose-response for drug sensitivity such as IC50 and not investigate biological response or affected pathways [48].

One limitation of these approaches is the lack of easy access for wider use, for example, in the form of a web application. DoseRider allows direct modeling of the entire pathway, preserving gene correlations and providing a more biologically meaningful dose-response curve. The combination of mixed models with cubic splines offers flexibility in capturing complex dose-response patterns beyond predefined functional classes. Additionally, with the development of an R package and a web tool, DoseRider ensures easy integration and accessibility.

## 4 Conclusion

In this study we presented DoseRider, an easy-to-use web application and a companion R package designed for multi-omics dose-response analysis at the level of biological pathways or signatures using generalized mixed effect models. It allows users to fit non-linear dose-response models and detect significantly affected pathways. Custom or provided multi-omics data such as RNA sequencing or metabolomics data can be analyzed and annotation of a collection of pathways and gene sets from various species were provided. The usability of DoseRider was demonstrated by a use case based on analyses of RNA sequencing data, but the framework is flexible to analyze other data types such as metabolites. This powerful package streamlines the integration of dose-response studies into research workflows and offers various visualization options. DoseRider is also able to assess various thresholds such as effective concentrations or benchmark doses (BMD) or trend change doses (TCD) for regulatory risk assessment.

In conclusion, DoseRider provides a powerful efficient solution for the challenges associated with toxicogenomis, studying perturbation effects, and analyzing molecular response of compounds.

## Funding

This research was funded in whole or in part by the Austrian Science Fund (FWF) [grant doi 10.55776/I6122 to JMG] and the Austrian Research Promotion Agency (FFG) [grant no. 864710 to JMG]. For open access purposes, the authors applied a CC BY public copyright license to any author accepted manuscript version arising from this submission.

## CRediT authorship contribution statement

**Monfort-Lanzas Pablo:** Writing – review & editing, Writing – original draft, Visualization, Software, Methodology, Conceptualization. **Gostner Johanna Maria:** Writing – review & editing, Writing – original draft, Supervision, Resources, Investigation, Conceptualization. **Hackl Hubert:** Writing – review & editing, Writing – original draft, Supervision, Resources, Investigation, Conceptualization.

## Declaration of Generative AI and AI-assisted technologies in the writing process

During the preparation of this work the authors used DeepL to improve readability and language. After using this tool/services, the authors reviewed and edited the content as needed and took full responsibility for the content of the publication.

## Declaration of Competing Interest

The authors declare no conflict of interest.
