# Supplementary material for: Modeling omics dose-response at the pathway level with DoseRider
Source: Comput Struct Biotechnol J. 2025 Apr 3;27:1440–8. doi: 10.1016/j.csbj.2025.04.004 (PMC12001094; doi:10.1016/j.csbj.2025.04.004)
Supplement: Supplementary file 1 — Supplementary material [file mmc1.docx]

**Supplementary material**


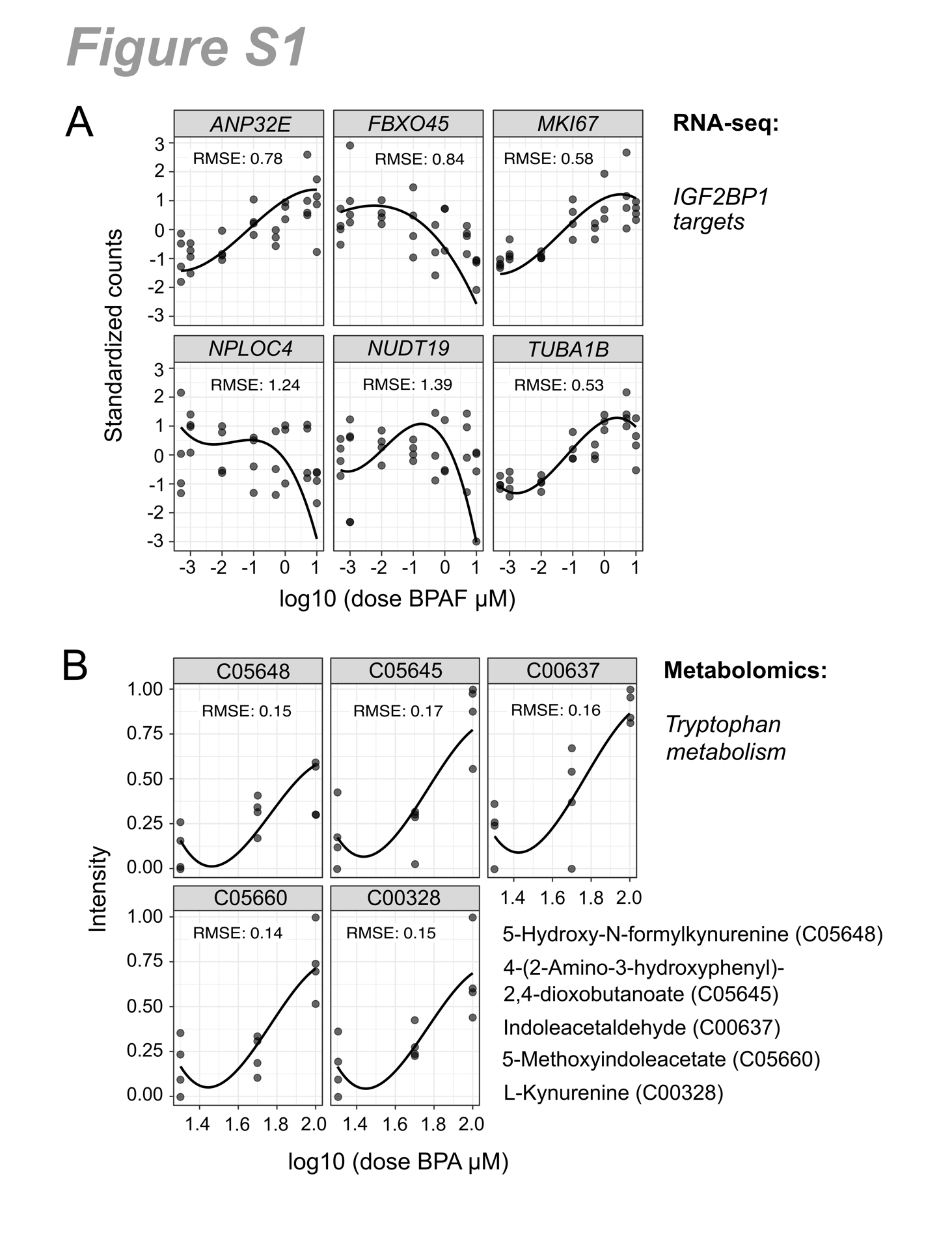


**Figure S1:** Comparison of predicted expression profiles of selected genes with normalized expression from biological replicates. As goodness-of-fit measure the root mean square errors (RMSE) are indicated.
